# Noise Annoyance in Urban Children: A Cross-Sectional Population-Based Study

**DOI:** 10.3390/ijerph13111056

**Published:** 2016-10-28

**Authors:** Natacha Grelat, Hélène Houot, Sophie Pujol, Jean-Pierre Levain, Jérôme Defrance, Anne-Sophie Mariet, Frédéric Mauny

**Affiliations:** 1 Centre Hospitalier Régional Universitaire de Besançon, Centre de Méthodologie Clinique, 2 place Saint Jacques, 25030 Besançon Cedex, France; nat1799@hotmail.fr (N.G.); sophie.pujol@univ-fcomte.fr (S.P.); 2 Laboratoire Chrono-Environnement, UMR 6249 Centre National de la Recherche Scientifique/Université de Bourgogne Franche-Comté, 2 place Saint Jacques, 25030 Besançon Cedex, France; 3 Laboratoire ThéMA, UMR 6049 Centre National de la Recherche Scientifique/Université de Bourgogne Franche-Comté, UFR Lettres SHS, 32 rue Mégevand, 25030 Besançon Cedex, France; helene.houot@univ-fcomte.fr; 4 Laboratoire de Psychologie EA 3188, 3 rue Mégevand, 25032 Besançon Cedex, France; jp.levain@orange.fr; 5 Division Acoustique Environnementale et Urbaine, Centre Scientifique et Technique du Bâtiment (CSTB), 24, rue Joseph Fourier, 38400 Saint-Martin-d’Hères, France; jerome.defrance@cstb.fr; 6 CHRU Dijon, Service de Biostatistique et d’Informatique Médicale (DIM), Université de Bourgogne, F-21000 Dijon, France; anne-sophie.mariet@chu-dijon.fr; 7 INSERM, CIC 1432, Dijon University Hospital, Clinical Investigation Center, Clinical Epidemiology/Clinical Trials Unit, F-21000 Dijon, France; 8 INSERM UMR 1181, Biostatistics, Biomathematics, Pharmacoepidemiology and Infectious Diseases (B2PHI), Université de Bourgogne, F-21000 Dijon, France

**Keywords:** children, noise annoyance, chronic noise exposure, urban area, social inequality

## Abstract

Acoustical and non-acoustical factors influencing noise annoyance in adults have been well-documented in recent years; however, similar knowledge is lacking in children. The aim of this study was to quantify the annoyance caused by chronic ambient noise at home in children and to assess the relationship between these children′s noise annoyance level and individual and contextual factors in the surrounding urban area. A cross sectional population-based study was conducted including 517 children attending primary school in a European city. Noise annoyance was measured using a self-report questionnaire adapted for children. Six noise exposure level indicators were built at different locations at increasing distances from the child′s bedroom window using a validated strategic noise map. Multilevel logistic models were constructed to investigate factors associated with noise annoyance in children. Noise indicators in front of the child′s bedroom (*p* ≤ 0.01), family residential satisfaction (*p* ≤ 0.03) and socioeconomic characteristics of the individuals and their neighbourhood (*p* ≤ 0.05) remained associated with child annoyance. These findings illustrate the complex relationships between our environment, how we may perceive it, social factors and health. Better understanding of these relationships will undoubtedly allow us to more effectively quantify the actual effect of noise on human health.

## 1. Introduction

The European Parliament Directive 2002/49/EC [1] defines environmental noise as an unwanted or harmful outdoor sound created by human activities, including noise emitted by road, rail, or aircraft traffic or industrial sites. Growing demand for air and road travel means that more people are being exposed to noise, a fortiori affecting more children. Noise from road transportation affects a large number of people: in the largest European cities (populations exceeding 250,000), data suggests that nearly 60 million people are exposed to long-term road traffic noise levels averaging in excess of 55 dBA L_den_ (weighted average day, evening, night) [2]. The World Health Organization recognizes noise as an important factor that may affect health [3,4]. The auditory effects of noise on adults have been well established [5]. According to Clark [6], there is convincing evidence of the non-auditory effects of noise on some aspects of adult health, such as sleep disturbances [7,8], hypertension and coronary heart disease [9,10] and a negative impact on cognition [6]. Noise also induces auditory effects in children; however, most of these effects are long-term and cumulative [11]. Children are less sensitive to sleep disturbances [8] but more sensitive to physiological effects such as blood pressure reactions [12]. The large-scale RANCH study (road traffic and aircraft noise exposure and children′s cognition and health: exposure-effect relationships and combined effects) showed that aircraft noise exposure could impair children′s cognitive development, especially in the area of reading comprehension [13].

Annoyance is one of the most widespread and well-documented responses to noise [14]. Annoyance can be defined as a feeling of discomfort [15] or a certain degree of long-term dissatisfaction, disturbance, or irritation with respect to the acoustic environment [16]. In some cases, annoyance may lead to stress responses, followed by symptoms and possibly illness. Strong annoyance caused by road traffic noise has been associated with significant and elevated risks of many diseases, such as cardiovascular problems, depression, migraines, and respiratory and arthritic symptoms [15]. An assessment of the dose-response relationship between noise exposure and annoyance showed that for an equivalent noise level, annoyance in adults varied according to the noise source [17]. Although high noise exposure has been associated with a high level of annoyance, sound level only partly explains the variance in the association between noise and annoyance in the population [18]. At most, approximately one third of this variance can be “explained” by acoustic factors, and another third can be explained by non-acoustic factors (personal or social) that affect perceptions of and attitudes towards noise [15,16,19]. Concerning acoustic factors, it has been hypothesized that noise characteristics affect the annoyance response, especially the number of noise events [20,21] and particularly traffic characteristics [22]. Among non-acoustic factors, individual noise sensitivity is one of the most widely accepted influencing factors [18,23]. Socioeconomic factors (especially educational level) [24,25], personal attitudes towards noise and its sources [26], and housing conditions [27] may also influence annoyance.

Children’s noise annoyance may differ from adults′ in several ways. Children are more exposed to noise than adults: they spend more time outdoors during daylight hours (such as on the way to school and on the playground) than adults do. Children are also more sensitive to noise than adults because they are in a critical developmental period [15] and have a less developed coping repertoire [28]. Although the consequences of annoyance on children′s health have been thoroughly assessed, a dose-response relationship has mostly been established in areas near international airports [29]. Outside of the airport context, few studies have focused on noise annoyance in children [30,31]. Concerning road traffic noise, Lercher et al. highlighted a difference in the dose-response curves between mothers and schoolchildren; they also identified the influence of contextual determinants such as physical, psychological, dispositional and social factors [31]. However, annoyance due to transportation and/or ambient noise in children living in urban areas has not yet been widely explored.

This study aimed to quantify annoyance at home caused by transportation and ambient noise in children and to assess the relationship between these children′s noise annoyance levels and individual or contextual factors in a medium-sized city.

## 2. Materials and Methods

### 2.1. Study Design and Participants

The study took place in the city of Besançon (117,000 inhabitants, France), a “medium sized” European city (i.e., a city of 100,000 to 500,000 inhabitants) [32]. This work was the second part of a multidisciplinary research programme conducted under the auspices of the National Program for Research and Innovation in Land Transport [33,34,35,36]. The eligibility criteria were as follows: 3rd grade of primary school in 2006–2007 and enrolled in one of the 35 public schools in Besançon. A self-administered, standardized three-part questionnaire was provided to the children′s families. Distribution and collection of the questionnaire were ensured by the teachers. The school offered help to families who did not speak the questionnaire language (French) at home. Hearing-impaired children were not included in this study. Children living outside of Besançon or not having lived in the same house for at least one year were excluded. Only one child per dwelling was considered for inclusion in this study. If more one eligible child lived in the same house, the study child was randomly selected. Data were collected during the spring of 2007.

### 2.2. Children′s Noise Annoyance

The intensity of noise annoyance was measured using standard questions adapted for children [37] and answered on a 4-point Likert scale: not at all, a little, moderately, and very much. Noise annoyance was evaluated for each of the six following sources, regardless of the time of day during which they were encountered: road traffic; rail traffic, shops/deliveries, bars/dance clubs, schools/playgrounds/athletic fields, and industrial/commercial areas. Three noise annoyance indices were defined as follows. Road traffic noise annoyance was first analysed independently. As suggested by Niemann [38], “general transport noise annoyance” was then defined by grouping annoyance from road and rail traffic noises. Finally, “ambient noise annoyance” referred to the cumulative annoyance resulting from all six noise source types. The last two indices were defined by taking the maximum annoyance score reported for each individual noise source. As suggested by Babsich et al. [30], these noise annoyance indices were then dichotomized as follows: not annoyed (Likert scale = not at all or a little) or annoyed (Likert scale = moderately or very much).

### 2.3. Noise Exposure Assessment

Children′s noise exposure was quantified using a validated strategic noise map developed by the team [33,34,39] in accordance with the European Commission‘s Environmental Noise Directive 2002/49/CE [1] and using the MITHRA-SIG v.2 noise-prediction software developed by Geomod and the French Scientific and Technical Centre for Building (CSTB). The following sources were modelled: road and rail traffic, pedestrian pathways, fountain basins, schoolyards and bus stops. The questionnaires were used to precisely locate each child’s dwelling (address, floor and type of dwelling) and the façade of the child′s bedroom (view from the child′s bedroom window and name of street in front).

For each child, two noise indices were calculated from the building façade at the floor-level of the child’s room: (i) in front of the child’s bedroom and (ii) on the façade most exposed to noise. These indices were expressed as the L_den_ (in dBA), a daily equivalent A weighted sound level (in decibels, dBA) with an addition of 5 dBA for the evening period and 10 dBA for the night period [1].

A buffer radius was also defined around each building, varying from 50 to 400 m [39]. For each buffer, the average noise level at 2 m above ground-level was computed and expressed as a daily equivalent A-weighted sound level, named L_Aeq, 24h_.

Finally, two official French zone scales (National Institute for Statistics and Economic Studies (INSEE)) were used: census blocks (size of an urban block) and census block groups (grouping together several adjacent census blocks, with between 1800 and 5000 inhabitants) [40]. For each zone, daily average noise levels L_Aeq, 24h_ were calculated.

### 2.4. Potential Influential Factors

Characteristics of the child (age and sex), his or her family (family size, number of people living together, and main language spoken at home) and their dwelling (dwelling type, type of built surroundings, window type, view from the child′s bedroom window, and how long the family has lived in this dwelling) were collected.

In addition, the family′s satisfaction with their dwelling and its environment were evaluated using a numerical scale from 1 to 5. Satisfaction variables were dichotomized, putting the highest category, defined as “very satisfied”, in opposition to a class combining the “not at all” through “moderately satisfied” categories.

Family socioeconomic status was defined using parental occupation and employment status. Four socioeconomic status classes were determined according to the INSEE classifications as follows: socio-economic status (SES) SES-1 = labourers, unemployed, or non-working; SES-2 = non-managerial position or clerk; SES-3 = mid-level employment or middle management position; and SES-4 = senior management, craftsman, shopkeeper, business owner, or corporate manager. The household′s socioeconomic status was considered to be that of the more privileged member of the couple. The parents′ employment status was used to determine if the family had at least the equivalent of one full-time worker in the family (one parent full-time or two parents working part-time). The other included socioeconomic characteristics were overcrowding (average people per room over one) and parental educational levels. We also included eight neighbourhood contextual socioeconomic characteristics: percentage of blue-collar workers in the labour force, percentage of foreigners in the total population, percentage of immigrants in the total population, percentage of single-parent families, percentage of employed people in the labour force, percentage of household labour, percentage of owner-occupied primary residences and percentage of households without a car. These characteristics have been previously selected to assess deprivation and were defined at the level of the census block groups (using the 2009 INSEE database) [41].

The urban environment was characterized at the census block level by build-up pattern, built density, and human land use [42]. Three urban environments were defined: (i) mixed residential area, individual housing and activity centre; (ii) densely urbanized area; and iii) social housing.

### 2.5. Statistical Analysis

The dataset was hierarchically organized into four levels: child, building, census block, and census block group. The associations between reported noise annoyance and individual and contextual factors were assessed using multilevel logistic regression models. To investigate the factors independently associated with moderate to severe annoyance, each variable was individually introduced into a “null” model (univariate analyses). For the second step, backward selection was applied to the subset of variables with a *p*-value of 0.20 or less in the univariate analyses. This backward stepwise selection procedure was run at each level, starting from the lowest to the highest and using a Restricted Iterative Generalized Least Square (RIGLS) algorithm. An equivalent Bayesian model was then created by incorporating the prior distributions of each of the parameters in the model and determining the resulting posterior distributions. We ran a burn-in of 500 iterations before the parameter′s values were stored during the following 500,000 iterations. The diagnosis (based on chain history, posterior density plots, and Raftery-Lewis diagnosis) provided no reason to suspect lack of convergence [43]. Odds ratios and 95% credibility intervals were then calculated. To explore the residual spatial connections between areas (contiguity, proximity), a conditional autoregressive (CAR) structure was introduced into the final models at the census block group level. The distribution of each spatial effect was centred on the mean of its neighbours. In practice, the definition of a neighbourhood was based on adjacent location: all census block groups sharing a border with a census block group of interest were considered “neighbours”. Models were compared using the Deviance Information Criterion (DIC) diagnosis, a generalization of the Akaike′s Information Criterion. A missing value category was assigned to subjects having no available value for potentially influential factors unless the frequency of the missing value was over 10% or the missing value′s distribution was not random in the univariate analysis. The threshold for statistical significance was set at 0.05. Two digits were retained and if necessary a “10^−3^” was used for *p*-values. The MLwiN 2.24 (University of Bristol, Bristol, UK) software program was used to perform these analyses [44].

### 2.6. Ethics

This study was approved by the French National Committee for the Treatment of Information in Health Research (CCTIRS) and by the French National Computing and Freedom Committee (CNIL) (NO.118.23.59, 13 July 2006). Written consent was obtained.

## 3. The Results

From the 964 identified schoolchildren from Besançon, 746 (77.4%) questionnaires were collected, and 654 children were eligible. Five hundred and seventeen annoyance questionnaires were returned by eligible children. The main characteristics of the study′s subjects, households, and neighbourhoods are presented in Table 1. The age of the children ranged between 7 and 11 years, and the mean age was 8.1 years (standard deviation = 0.4). Children′s noise exposure characteristics may be found in Table 2. Contextual socioeconomic characteristics defined at the census block group level are exhibited in Appendix Appendix Table A1.

Non-respondents to the third part of the questionnaire (concerning annoyance, *n* = 137) did not differ significantly from respondents in either the family′s residential satisfaction (*p* ≥ 0.32) or outdoor noise levels measured in front of the most exposed façade of the child’s home (*p* = 0.31). Compared with respondents, non-respondents more often lived in social housing, were more often surrounded by buildings or busy streets, and had lower educational and socioeconomic levels and a higher outdoor noise level in front of the child′s bedroom (all *p* < 0.02).

Among the 517 children analysed, 179 (34.6%) were annoyed by road traffic noise, 197 (38.1%) were annoyed by general transportation noise and 316 (61.1%) were annoyed by ambient noise. In the bivariate analyses of the three noise annoyance indices, neither family characteristics (all *p* ≥ 0.53) nor age or sex (all *p* ≥ 0.73) were significantly associated with child annoyance. Dwelling type, type of built surroundings and view from the child′s bedroom window significantly differed by annoyance status (all *p* ≤ 0.08, *p* ≤ 0.10 and *p* ≤ 0.01, respectively). Family residential satisfaction was significantly associated with child annoyance (all *p* < 10^−3^). All socioeconomic characteristics defined at the family and the neighbourhood level were significantly associated with child annoyance (all *p* ≤ 0.01). Child annoyance caused by road traffic noise, general transportation noise or ambient noise was significantly associated with most of the noise indicators (Table 3). However, as these indicators were assess at greater distances from the children′s bedrooms, these associations became less (or no longer) significant. After adjustment for the noise indicator assessed in front of the child′s bedroom, others indicators were no longer significant (all *p* > 0.06).

In the multivariate analysis, child annoyance caused by road traffic noise, general transportation noise and ambient noise remained significantly associated with noise level in front of the child′s bedroom (all *p* ≤ 0.01), family residential satisfaction (all *p* ≤ 0.03) and socioeconomic characteristics of individuals and their neighbourhood (all *p* ≤ 0.05) (Table 4, Table 5 and Table 6, respectively).

A comparison of models with and without spatial correlation (CAR model) did not provide an argument to adjust for spatial correlation: the DICs associated with CAR models were equal to or higher than the DICs associated with the other models, and parameter estimates were comparable. Final results were retained from the non-spatially structured Bayesian multilevel model. For the three types of noise annoyance, the DIC associated with the models including or not including a conditional autoregressive structure were 577.6 vs. 576.1, 615.2 vs. 615.1 and 665.4 vs. 669.3, respectively. Considering the special relationship between noise annoyance and residential satisfaction, a complementary multivariate analysis was performed without inclusion of the residential satisfaction variable. For the three types of noise annoyance, the results were very similar to the results presented below, especially the coefficient values associated with outdoor noise level in front of the child′s bedroom.

## 4. Discussion

In this study, noise annoyance in children was associated with noise exposure indicators in front of children′s bedrooms, family residential satisfaction and the socioeconomic characteristics of individuals and their neighbourhood.

In this population-based study, the questionnaire response rate was high (77.4%). However, only public schools were included because of the methodology of the first part of the research programme (private schools were not systematically included in the national standardized assessment of the French Ministry of the National Education). However, the results obtained from the study children were equivalent to those obtained at the national level [35]. The socio demographic characteristics of the city of Besancon were very close to those of the French cities with 100,000 to 200,000 inhabitants in 2010: the percentage of 0–14 years old were 15% vs. 18.5%, the manager percentages were 10% vs. 7% and the percentage of retired persons were 23% vs. 24%, respectively [45]. Non-respondent families mainly differ from respondent families in average socioeconomic status (lower in non-respondents) and noise exposure indices (higher in non-respondents), which were factors positively associated with a higher reported level of annoyance. The possible consequences of this difference could be moderate underestimation of the annoyance rates and estimated odds ratio values.

There has been no consensus on which instrument should be used for investigating noise annoyance in children. Some authors have used a 4-point Likert scale [46], and other authors have used a 5-point Likert scale [29]. A 4-point scale was likely to be less complex and more appropriate for inclusion in this population-based survey of primary schoolchildren using a self-administered questionnaire at home. Finally, to account for potential difficulties in answering on a graduated scale in children, annoyance responses were dichotomized [30]. In a medium-sized city where the population was not exposed to aircraft or highway traffic noise, grouping moderate and severe annoyance classes appeared relevant to studying the reported annoyance level. A validated noise map was used to quantify chronic noise exposure independent from annoyance status (and other variables) and to avoid a differential measurement bias. Multilevel modelling was applied according to the complex hierarchical structure of the data.

This study compared noise exposure indicators assessed at different locations at increasing distances from the child′s bedroom window. In this way, a closer association between the level of noise annoyance and the most representative indicator of the level of outdoor noise directly outside the child′s bedroom was identified. Children spend lot of time in their bedrooms when they are at home [47], and they are likely to have more control over their immediate indoor soundscape and a sharper perception of outdoor environmental noise. The relationship between outdoor and indoor noise is complex and modulated by numerous factors [34]. Independent from the indoor noise level and despite attenuation provided by the buildings, the outdoor noise perceived by children in their bedrooms remained a substantial provider of noise annoyance. Our results also support the idea that outdoor noise exposure at the child′s bedroom façade should be considered when exploring noise consequences on children.

Noise annoyance in children was associated with dwelling and/or neighbourhood satisfaction. The degree of residential satisfaction reported by parents is likely shared by other family members and particularly by children who are approximately 8 years old. Children use the reactions of the adults in their family as models for their own reactions [48]. Although an association between residential satisfaction and noise annoyance has been demonstrated in adults [26], this is to our knowledge the first time that similar results have been found in children. We caution, however, that the direction of this relationship’s causality has yet to be established: some authors have found that noise annoyance has a strong negative effect on overall residential satisfaction [49].

Noise exposure level is known to be associated with socioeconomic level. Evans has described a “so-called” environmental injustice—the fact that people with lower socioeconomic status are more exposed to multiple adverse environmental conditions, including high noise levels [50]. After adjustment for noise exposure levels, several socioeconomic factors included in this study (regardless of whether they were defined at the individual or neighbourhood level) remained significantly associated with the noise annoyance in children. These findings are consistent with those of Babisch′s study [30]: children of lower socio-economic status were more often annoyed by road traffic noise than children of medium and high socioeconomic status. Conversely, in adults, this relationship has been established as positive [24,51]. In a similar vein, the association between annoyance caused by road traffic in children and urban environment was consistent with the results of the study conducted by Lercher et al., which demonstrated the influence of social and physical context on noise annoyance [31]. Independent of residential satisfaction, these findings might reveal an indirect influence of noise annoyance on well-being and life satisfaction [49].

Several points should be discussed. Noise sensitivity has been shown to have an effect on the judgement of annoyance [23]. Sensitivity is a stable personality trait [52] that influences attitudes towards environmental noise in general [14,22]. Unfortunately, these data did not allow for an analysis of the influence of such non-acoustic factors. Miedema et al. found that the influence of noise sensitivity on noise annoyance becomes especially important at higher exposure levels [53]. No particularly noisy infrastructures, such as airports or highways, are present in the residential areas of Besançon. The main noise source in this area is ground transportation. Therefore, not analysing sensitivity likely did not greatly influence our study results. Considering the large sample size, differences in noise annoyance caused by noise sensitivity might also have averaged out within the analysis. Although the association between annoyance in children and outdoor noise level in front of their bedroom has been demonstrated in this study, the actual chronic noise exposure was not assessed. It partially depends on whether the window of the bedroom is usually open or closed. No question about such habits was included in the questionnaire so there is still a lack of knowledge about the influence of this behavioural factor. Nevertheless, outdoor indicators are often used as a proxy to summarize the overall outdoor and indoor environmental exposure [54]. Some specific noise sources, especially powered two-wheelers, are known to induce high levels of noise annoyance [42]; however, for technical reasons (no available measurements of two-wheeler traffic or punctual emerging events), these noise sources were not considered in the noise mapping process. This is a common limitation of noise mapping assessments. The cross-sectional design of this study allowed for the examination of statistical associations but did not consider temporal sequences. Therefore, this study design precluded causal inference [49]; the direction of the causation between the associated events, as illustrated here by the association between noise annoyance and residential satisfaction, should be discussed very cautiously.

## 5. Conclusions

In conclusion, in the context of a high-risk group, we have highlighted the influence of acoustical and non-acoustical factors on noise annoyance in children residing in middle-sized urban residential areas. The findings illustrate the complex relationships between our environment, how we may negatively (or positively) perceive it, the influence of social factors on this perception and its consequences on health. Better understanding of these associations will undoubtedly allow us to more effectively quantify the actual effect of noise on human health.

## Figures and Tables

**Table 1:** Participant characteristics defined at the child and census block levels (*n* = 517).

Participant Characteristics	*n* (%)
**Individual characteristics**	
Sex	
Boys	239 (46.3)
Girls	277 (53.7)
**Dwelling characteristics**	
Type of dwelling	
Apartment building	127 (24.7)
Detached or semi-detached house	388 (75.3)
Type of window	
Single-glazed	99 (19.2)
Double-glazed	397 (76.8)
View from the child’s bedroom window	
Courtyard	109 (22.2)
Grassy area	176 (35.9)
Low traffic street	112 (22.9)
Heavy traffic street	93 (19.0)
Type of built surroundings	
Apartment building only	192 (37.5)
Detached house only	93 (18.1)
Both apartment building and detached house	228 (44.4)
**Family residential satisfaction**	
Satisfaction with the dwelling	
Not to moderately satisfied	296 (70.3)
Very satisfied	125 (29.7)
Satisfaction with the environment	
Not to moderately satisfied	339 (68.8)
Very satisfied	154 (31.2)
**Socio-economic characteristics**	
Maternal education level	
Elementary and middle school	92 (18.9)
High school	174 (35.6)
University	222 (45.5)
Paternal education level	
Elementary and middle school	91 (22.8)
High school	118 (29.6)
University	190 (47.6)
Parents′ employment status	
No full-time worker	101 (19.7)
At least one full-time worker or both part time	412 (80.3)
Household socio-economic status ^a^	
SES-1	46 (9.2)
SES-2	149 (29.9)
SES-3	144 (28.9)
SES-4	159 (32.0)
Overcrowding	
No	379 (76.3)
Yes	118 (23.7)
**Urban environment**	
Densely urbanized area	77 (14.9)
Social Housing	142 (27.5)
Mixed Residential Area, Individual Housing or Activity Centre	298 (57.6)

^a^ SES-1 = labourers, unemployed, non-working; SES-2 = non-managerial position or clerk; SES-3 = mid-level employment or middle management position; SES-4 = senior management, craftsman, shopkeeper, business owner, corporate manager. Missing data: age (*n* = 50), sex (*n* = 1), type of dwelling (*n* = 2), type of window (*n* = 21), view from child′s bedroom window (*n* = 27), type of built surroundings (*n* = 4),parent′s satisfaction with the dwelling (*n* = 96), parent′s satisfaction with the environment (*n* = 24), maternal education level (*n* = 29), paternal education level (*n* = 118), parent′s employment status (*n* = 4), household socio-economic status (*n* = 19), overcrowding (*n* = 20), urban environment (*n* = 0).

**Table 2:** Outdoor noise exposure level at the child, building, census block and the census block group levels (*n* = 517).

Noise Exposure in dBA	Mean (sd ^a^)	Range
**Child level ^b^**		
In front of child’s bedroom	56.4 (4.5)	44–69
In front of the most exposed façade	59.2 (4.0)	47–69
**Building level ^c^**		
Buffer 50 m average	51.0 (5.6)	35–66
Buffer 400 m average	55.5 (3.7)	41–62
**Census block level ^c^**		
Census blocks average	53.2 (5.1)	39–60
Census block groups level ^c^		
Census blocks group average	55.3 (3.6)	47–63

^a^ standard deviation; ^b^ L_den_; ^c^ L_Aeq, 24h_.

**Table 3:** Associations between annoyance related to road traffic noise, general transportation noise or ambient noise and the outdoor noise exposure indicators in children: bivariate analysis.

Outdoor Noise Exposure Indicators	Road Traffic Noise			General Transport Noise			Ambient Noise		
	OR ^a^	95% CI ^b^	*p*-Value	OR	95% CI	*p*-Value	OR	95% CI	*p*-Value
Noise level in front of child’s bedroom	2.83	1.84–4.35	<10^−3^	2.72	1.80–4.10	<10^−3^	1.84	1.24–2.72	2.10^−3^
Noise level in front of most exposed façade	2.53	1.44–4.47	10^−3^	2.25	1.30–3.90	3.10^−3^	1.39	0.84–2.32	0.22
Buffer 50 m noise average	2.27	1.56–3.29	<10^−3^	2.25	1.55–3.26	<10^−3^	1.40	0.97–2.04	0.07
Buffer 400 m noise average	2.44	1.28–4.65	7.10^−3^	2.77	1.45–5.30	2.10^−3^	1.05	0.60–1.86	0.85
Census block noise average	2.14	1.36–3.36	0.02	2.48	1.61–3.82	10^−3^	1.55	0.97–2.30	0.06
Census block groups noise average	2.51	1.29–4.89	0.03	2.69	1.34–5.45	6.10^−3^	1.19	0.66–2.13	0.56

^a^ Odds ratio associated with increase of 10 dBA; ^b^ 95% Confidence Interval.

**Table 4:** Annoyance caused road traffic noise in children: multivariate analysis.

Independent Variables	OR ^a^	95% CI ^b^	*p*-Value
Intercept	0.20	0.12–0.33	
**Child level**			
Satisfaction with the environment			0.02
Very satisfied	1.00		
Not to moderately satisfied	2.22	1.30–3.78	
Missing data	3.75	1.19–11.89	
Outdoor noise level in front of child’s bedroom ^c^	3.56	2.10–6.04	<10^−3^
**Census block level**			
Urban environment			0.01
Mixed Residential Area, Individual Housing and Activity Centre	1.00		
Densely Urbanized Area	1.58	0.80–3.13	
Public Housing	2.95	1.66–5.25	

^a^ Odds Ratio; ^b^ 95% Credibility Interval; ^c^ OR associated with an increase of 10 dBA.

**Table 5:** Annoyance caused by general transportation noise in children: multilevel analysis.

Independent Variables	OR ^a^	95% CI ^b^	*p*-Value
Intercept	0.27	0.07–0.89	
**Child level**			
Household socio economic status ^c^			0.04
SES-4	1.00		
SES-3	1.26	1.08–1.47	
SES-2	2.10	1.35–3.28	
SES-1	2.08	1.29–3.35	
Satisfaction with the dwelling			0.03
Very satisfied	1.00		
Not to moderately satisfied	2.30	1.33–3.98	
Missing data	2.10	1.29–3.43	
Outdoor noise level in front of child′s bedroom ^d^	2.51	1.42–4.43	<10^−3^

^a^ Odds Ratio; ^b^ 95% Credibility Interval; ^c^ SES-1 = labourers, unemployed, non-working; SES-2 = non-managerial position or clerk; SES-3 = mid-level employment or middle management position; SES-4 = senior management, craftsman, shopkeeper, business owner, corporate manager; ^d^ OR associated with an increase of 10 dBA.

**Table 6:** Annoyance caused by ambient noise in children: multivariate analysis.

Independent Variables	OR ^a^	95% CI ^b^	*p*-Value
Intercept	0.77	0.51–1.16	
**Child level**			
Satisfaction with the dwelling			7.10^−3^
Very satisfied	1.00		
Not to moderately satisfied	2.76	1.68–4.55	
Missing data	1.76	0.91–3.38	
Outdoor noise level in front of child’s bedroom ^c^ (10 dBA)	1.97	1.23–3.16	0.01
**Census block groups level**			
Percentage of households without a car (for an increase of 10%)	1.26	1.06–1.50	0.03

^a^ Odds Ratio; ^b^ 95% Credibility Interval; ^c^ OR associated with an increase of 10 dBA.

## Appendix A

**Table A1:** Contextual socioeconomic characteristics defined at the census block groups level (*n* = 517).

Contextual Socio-Economic Variables (Percentages)	Mean (Standard Deviation)	Range
Immigrants	12.6 (8.1)	3.2–28.7
Household labour	16.8 (8.4)	4.7–34.0
Single parents families	21.6 (9.1)	8.7–40.7
Employed people in the labour force	15.5 (8.0)	1.1–34.9
Blue-collar workers in the labour force	24.4 (11.6)	7.4–49.6
Owner-occupied primary residences	35.5 (19.1)	0.0–79.6
Households without a car	28.5 (12.5)	3.1–54.1

## References

[1] Directive 2002/49/EC of the European Parliament and of the Council of 25 June 2002 Relating to the Assessment and Management of Environmental Noise. http://faolex.fao.org/cgi-bin/faolex.exe?rec_id=029920&database=faolex&search_type=link&table=result&lang=eng&format_name=@ERALL.

[2] Vicente A.S. (2011). Laying the Foundations for Greener Transport. TERM 2011: Transport Indicators Tracking Progress towards Environmental Targets in Europe, EEA Report 7/2011.

[3] Guidelines for Community Noise. http://www.who.int/docstore/peh/noise/guidelines2.html.

[4] Burden of Disease from Environmental Noise. http://www.euro.who.int/__data/assets/pdf_file/0008/136466/e94888.pdf.

[5] Job R.F.S., Hatfield J. (2000). Effective Communication of Health Messages regarding Noise-Induced Health Effects. Noise Health.

[6] Clark C., Stansfeld S.A. (2007). The Effect of Transportation Noise on Health and Cognitive Development: A Review of Recent Evidence. Int. J. Comp. Psychol..

[7] Miedema H.M., Vos H. (2007). Associations between self-reported sleep disturbance and environmental noise based on reanalyses of pooled data from 24 studies. Behav. Sleep Med..

[8] Öhrström E., Hadzibajramovic E., Holmes M., Svensson H. (2006). Effects of road traffic noise on sleep: Studies on children and adults. J. Environ. Psycchol..

[9] Babisch W., Swart W., Houthuijs D., Selander J., Bluhm G., Pershagen G., Dimakopoulou K., Haralabidis A.S., Katsouyanni K., Davou E. (2012). Exposure modifiers of the relationships of transportation noise with high blood pressure and noise annoyance. J. Acoust. Soc. Am..

[10] Liu C., Fuertes E., Tiesler C.M.T., Birk M., Babisch W., Bauer C.P., Koletzko S., Heinrich J. (2013). The association between road traffic noise exposure and blood pressure among children in Germany: The GINIplus and LISAplus studies. Noise Health.

[11] Bistrup M.L., Babisch W., Stansfeld S.A., Sulkowski W. (2006). PINCHE’s policy recommendations on noise: How to prevent noise from adversely affecting children. Acta Paediatr..

[12] Babisch W., Houthuijs D., Pershagen G., Cadum E., Katsouyanni K., Velonakis M., Dudley M.L., Marohn H.D., Swart W., Breugelmans O. (2009). HYENA Consortium. Annoyance due to aircraft noise has increased over the years—Results of the HYENA study. Environ. Int..

[13] Stansfeld S.A., Berglund B., Clark C., Lopez-Barrio I., Fischer P., Ohrström E., Haines M.M., Head J., Hygge S., van Kamp I. (2005). Aircraft and road traffic noise and children′s cognition and health: A cross-national study. Lancet.

[14] Van Kamp I., Job R.S., Hatfield J., Haines M., Stellato R.K., Stansfeld S.A. (2004). The role of noise sensitivity in the noise-response relation: A comparison of three international airport studies. J. Acoust. Soc. Am..

[15] WHO LARES Final Report: Noise Effects and Morbidity. http://www.euro.who.int/__data/assets/pdf_file/0015/105144/WHO_Lares.pdf.

[16] Guski R. (1999). Personal and social variables as co-determinants of noise annoyance. Noise Health.

[17] Miedema H.M., Oudshoorn C.G. (2001). Annoyance from transportation noise: Relationships with exposure metrics DNL and DENL and their confidence intervals. Environ. Health Perspect..

[18] Gille L.A., Marquis-Favre C., Morel J. (2016). Testing of the European Union exposure-response relationships and annoyance equivalents model for annoyance due to transportation noises: The need of revised exposure-response relationships and annoyance equivalents model. Environ. Int..

[19] Ouis D. (2001). Annoyance from road traffic noise: A review. J. Environ. Psychol..

[20] Björkman M. (1991). Community noise annoyance: Importance of noise levels and the number of noise events. J. Sound Vib..

[21] Jakovljevic B., Paunovic K., Belojevic G. (2009). Road-traffic noise and factors influencing noise annoyance in an urban population. Environ. Int..

[22] Paunović K., Jakovljević B., Belojević G. (2009). Predictors of noise annoyance in noisy and quiet urban streets. Sci. Total Environ..

[23] Job R.F.S. (1988). Community response to noise: A review of factors influencing the relationship between noise exposure and reaction. J. Acoust. Soc. Am..

[24] Passchier-Vermeer W., Passchier W.F., Nicolopoulou-Stamati P., Hems L., Lammar P., Howard C.V. (2005). Environmental Noise, Annoyance and Sleep Disturbance. Environmental Health Impacts of Transport and Mobility.

[25] Van Renterghem T., Botteldooren D. (2012). Focused study on the quiet side effect in dwellings highly exposed to road traffic noise. Int. J. Environ. Res. Public Health.

[26] Lam K.C., Chan P.K., Chan T.C., Au W.H., Hui W.C. (2009). Annoyance response to mixed transportation noise in Hong Kong. Appl. Acoust..

[27] Fields J.M. (1993). Effect of personal and situational variables on noise annoyance in residential areas. J. Acoust. Soc. Am..

[28] Bistrup M.L. (2003). Prevention of adverse effects of noise on children. Noise Health.

[29] Van Kempen E., van Kamp I., Stellato R.K., Lopez-Barrio I., Haines M.M., Nilsson M.E., Clark C., Houthuijs D., Brunekreef B., Berglund B. (2009). Children′s annoyance reactions to aircraft and road traffic noise. J. Acoust. Soc. Am..

[30] Babisch W., Schulz C., Seiwert M., Conrad A. (2012). Noise Annoyance as Reported by 8- to 14-Year-Old Children. Environ. Behav..

[31] Lercher P., Brauchle G., Kofler W., Widmann U., Meis M. The Assessment of Noise Annoyance in Schoolchildren and Their Mothers. Proceedings of the 29th International Congress and Exhibition on Noise Control Engineering.

[32] Boddy M. (1999). Geographical Economics and Urban Competitiveness: A Critique. Urban Stud..

[33] Pujol S., Berthillier M., Defrance J., Lardies J., Petit R., Houot H., Levain J.P., Masselot C., Mauny F. (2012). Urban ambient outdoor and indoor noise exposure at home: A population-based study on schoolchildren. Appl. Acoust..

[34] Pujol S., Berthillier M., Defrance J., Lardies J., Levain J.P., Petit R., Houot H., Mauny F. (2014). Indoor noise exposure at home: A field study in the family of urban schoolchildren. Indoor Air.

[35] Pujol S., Levain J.P., Houot H., Petit R., Berthillier M., Defrance J., Lardies J., Masselot C., Mauny F. (2014). Association between ambient noise exposure and school performance of children living in an urban area: A cross-sectional population-based study. J. Urban Health.

[36] Levain J.P., Mauny F., Pujol S., Petit R., Houot H., Defrance J., Lardies J., Berthillier M. (2015). Exposition au bruit et performance scolaire des élèves de CE_2_. Psychol. Fr..

[37] Fields J., de Jong R., Flindell I., Gjestland T., Job R.F.S., Kurra S., Schuemer-Kohrs A., Vallet M., Yano T. Recommendation for Shared Annoyance Questions in Noise Annoyance Surveys. Proceedings of the 7th International Congress on Noise as a Public Health Problem.

[38] Niemann H., Bonnefoy X., Braubach M., Hecht K., Maschke C., Rodrigues C., Robbel N. (2006). Noise-induced annoyance and morbidity results from the pan-European LARES study. Noise Health.

[39] Tenailleau Q.M., Bernard N., Pujol S., Houot H., Joly D., Mauny F. (2015). Assessing residential exposure to urban noise using environmental models: Does the size of the local living neighborhood matters?. J. Expo. Sci. Environ. Epidemiol..

[40] National Institute of the Statistics and Economic Studies (INSEE). http://www.insee.fr/en/methodes/default.asp?page=definitions.htm.

[41] Lalloué B., Monnez J.M., Padilla C., Kihal W., Meur N.L., Zmirou-Navier D., Deguen S. (2013). A statistical procedure to create a neighborhood socioeconomic index for health inequalities analysis. Int. J. Equity Health.

[42] Houot H. Geographical approach of annoyance due to noise transportation. Proceedings of the 29th International Congress and Exhibition on Noise Control Engineering.

[43] Browne W.J., Rasbash J. (2009). MCMC Estimation in MLwiN.

[44] Rasbash J., Charlton C., Browne W.J., Healy M., Cameron B. (2009). MLwiN Version 2.1.

[45] Baccaïni B., Mattei M.F., Pumain D. (2015). Croissance démographique et changements sociaux dans les grandes unités urbaines françaises entre 1999 et 2010. Données Urbaines 7.

[46] Haines M.M., Stansfeld S.A., Brentnall S., Head J., Berry B., Jiggins M., Hygge S. (2001). The West London Schools Study: The effects of chronic aircraft noise exposure on child health. Psychol. Med..

[47] Xue J., McCurdy T., Spengler J., Özkaynak H. (2004). Understanding variability in time spent in selected locations for 7–12-year old children. J. Expo. Sci. Environ. Epidemiol..

[48] Van Kempen E., van Kamp I., Nilsson M., Lammers J., Emmen H., Clark C., Standfeld S. (2010). The role of annoyance in the relation between transportation noise and children′s health and cognition. J. Acout. Soc. Am..

[49] Urban J., Maca V. (2013). Linking Traffic Noise, noise Annoyance and Life Satisfaction: A Case Study. Int. J. Environ. Res. Public Health.

[50] Evans G.W., Kantrowitz E. (2002). Socioeconomic status and health: The potential role of environmental risk exposure. Annu. Rev. Public Health.

[51] Miedema H.M.E., Vos H. (1999). Demographic and attitudinal factors that modify annoyance from transportation noise. J. Acoust. Soc. Am..

[52] Stansfeld S.A. (1992). Noise, noise sensitivity and psychiatric disorder: Epidemiological and psychophysiological studies. Psychol. Med..

[53] Miedema H.M.E., Vos H. (2003). Noise sensitivity and reactions to noise and other environmental conditions. J. Acoust. Soc. Am..

[54] Nieuwenhuijsen M., Paustenbach D., Duarte-Davidson R. (2006). New developments in exposure assessment: The impact on the practice of health risk assessment and epidemiological studies. Environ. Int..

